# Superiority of dendritic cell vaccine vs tumor cell vaccine: survival by stratification subsets in MACVAC randomized Phase II trial of patient-specific vaccines utilizing antigens from autologous melanoma tumor cell lines

**DOI:** 10.1186/2051-1426-3-S2-P202

**Published:** 2015-11-04

**Authors:** Robert O Dillman, Edward McClay, Thomas Amatruda, George Semeniuk, Clark Haskins, Robert Weber, David Burtzo, Carol DePriest, Denysha Carbonell, Andrew Cornforth

**Affiliations:** 1 Caladrius BioSciences, Inc, Irvine, CA, USA; 2 California Cancer Associates for Research and Excellence (cCARE), Institute for Melanoma Research & Education, Encinitas, CA, USA; 3 Minnesota Oncology, Fridley, MN, USA; 4 Orange Coast Oncology and Hematology, Newport Beach, CA, USA; 5 New Mexico Cancer Center, Albuquerque, NM, USA; 6 St. Mary's Medical Center and Sutter Health, San Francisco, CA, USA; 7 Pacific Shores Medical Group, Huntington Beach, CA, USA; 8 Cancer Biotherapy Research Group, Franklin, TN, USA

## 

In a randomized Phase II trial conducted in patients with metastatic melanoma, superior overall survival (p=0.007) was observed for 18 patients treated with vaccines that consisted of autologous dendritic cells loaded with antigens from irradiated autologous melanoma stem cells, (DC-TC, aka eltrapuldencel-T, NBS20 and CBLS20) compared to 24 patients treated with vaccines consisting of autologous irradiated melanoma stem cells (TC) [ClinicalTrials.gov NCT00436930].[1] Both vaccines were admixed with GM-CSF as an adjuvant. Tumor cell lines that served as the source of patient-specific tumor-associated antigens were derived from metastases resected from patients with stage IV or recurrent stage III melanoma. The treatment schedule consisted of weekly subcutaneous injections for 3 weeks, and then monthly for 5 months. The current analysis was undertaken to determine the treatment effects of DC-TC vs TC in each of the subsets defined by the pre-randomization stratifications that were based on whether patients had measurable or non-measurable disease as defined by RECIST, and whether their most advanced stage of disease at the time of randomization had been stage IV or recurrent stage III disease. At the time of this analysis 5 DC-TC and 3 TC patients had been followed for 5 years; 5 patients (3 TC and 2 DC-TC) were alive but followed less than 5 years (minimum 3.5 years); 29 were deceased. No patients were lost to follow up. The survival results are summarized in Table 1. Although the numbers are small, DC-TC immunotherapy was associated with superior survival in each of the four different subsets defined by the stratification variables. Eltrapuldencel-T has moved forward into a pivotal Phase III trial sponsored by Caladrius BioSciences, Inc.

**Table 1:** 

Stratification	Treatment	# of patients	Median survival in months	3-year overall survival
Measurable	DC-TC	8	17.6	33%
Measurable	TC	9	6.5	11%
Non-Measurable	DC-TC	10	Not Reached	56%
Non-Measurable	TC	15	31.3	33%

Recurrent Stage III	DC-TC	3	Not Reached	67%
Recurrent Stage III	TC	6	30.3	17%
Stage IV	DC-TC	15	40.4	60%
Stage IV	TC	18	16.9	28%

## Trial registration

ClinicalTrials.gov identifier NCT00436930.
